# Supplementary material for: A semi-automated genetic screen using genome-wide association analysis identifies modulators of stress-induced sleep in Caenorhabditis elegans
Source: G3 (Bethesda). 2026 Sep 28;16(11):jkag266. doi: 10.1093/g3journal/jkag266 (PMC13732485; doi:10.1093/g3journal/jkag266)
Supplement: Supplementary file 1 [file jkag266_supplementary_data.zip › Supplemental_Material_Legends_G3-2026-407279.docx]

# Supplementary material

- **Movie S1. Robotic transfer of *C. elegans* from agar plate to WorMotel.** The live images shown on the left and right were recorded simultaneously by the low- and high-magnification cameras in the robotic system. The field of view (FOV) was 6.4 × 4.9 cm for low magnification and 1.88 × 1.57 mm for high magnification. The high-magnification FOV was located approximately at the center of the low-magnification FOV.
- **Table S1. Information on *C. elegans* strains used in this study.** The file contains two spreadsheets containing information for MMP stains and candidate mutant strains. Key information includes strain name, genotype, identifier, source, and sleep scores.
- **Table S2. Ranks of individual known sleep-regulating genes from the SKAT analysis.** The file contains two spreadsheets showing the ranks of individual known genes in the initial and expanded sets, respectively, from the SKAT analysis.
- **Table S3. List of ranked genes from the SKAT analysis with** $\boldsymbol{N}_{\boldsymbol{a}}$ **= 5.** The table contains genes represented by at least five non-synonymous alleles ($N_{a}$ = 5; Table 1) in the MMP screen, ranked by the statistical significance of their associations with the SIS phenotype in the SKAT analysis.
- **Table S4. List of ranked genes from the SKAT analysis with** $\boldsymbol{N}_{\boldsymbol{a}}$ **= 3.** The table contains genes represented by at least three non-synonymous alleles ($N_{a}$ = 3; Table 1) in the MMP screen, ranked by the statistical significance of their associations with the SIS phenotype in the SKAT analysis.
- **Software S1.** **Data and source code for reproducing the results**. The folder includes data files, MATLAB scripts for data processing, visualization, and statistical analysis, R scripts for SKAT analysis, and documentation.
